# Complete Genome Sequences of Three Laboratory Strains of Dengue Virus (Serotypes 2, 3, and 4) Available in South Korea

**DOI:** 10.1128/genomeA.01389-15

**Published:** 2015-11-25

**Authors:** Yun Young Go, Eunhye Jung, Udeni B. R. Balasuriya

**Affiliations:** a Virus Research and Testing Group, Division of Drug Discovery Research, Korea Research Institute of Chemical Technology, Daejeon, South Korea; b Maxwell H. Gluck Equine Research Center, Department of Veterinary Science, College of Agriculture, Food and Environment and Department of Microbiology, Immunology and Molecular Genetics, College of Medicine, University of Kentucky, Lexington, Kentucky, USA

## Abstract

Here, we report the complete genome sequences of three laboratory strains of dengue virus serotype-2 (DENV-2/KBPV-VR-29), -3 (DENV-3/KBPV-VR-30), and -4 (DENV-4/KBPV-VR-31) obtained from the Korea Bank for Pathogenic Viruses (http://kbpv.knrrc.or.kr). The complete genetic information presented in this study on commonly used DENV laboratory strains provides valuable information for future studies.

## GENOME ANNOUNCEMENT

Dengue virus (DENV) is an enveloped virus with a single-stranded, positive-sense RNA genome of approximately 10,700 nucleotides in length (1). The genomic RNA includes 5′- and 3′-untranslated regions and a single open reading frame that encodes a single polyprotein that is cleaved into three structural proteins (capsid [C], premembrane/membrane [prM/M], and envelope [E]) and seven nonstructural proteins (NS1, NS2A, NS2B, NS3, NS4A, NS4B, and NS5) (2). DENV comprises four distinct serotypes (DENV-1 to -4) that belong to the genus *Flavivirus* in the family *Flaviviridae* (3). Distinct genotypes have been identified within each serotype, highlighting the extensive genetic variability of the DENV serotypes. Dengue viruses cause a wide range of clinical symptoms in humans, from acute febrile illness dengue fever to life-threatening dengue hemorrhagic fever/dengue shock syndrome.

Our laboratory in the Division of Drug Discovery Research, Korea Research Institute of Chemical Technology, Daejeon, South Korea, has been routinely using three DENV serotypes for cell-based antiviral testing assays. However, the complete genome sequences of these viruses are unknown. This prompted us to sequence the complete genome of these three DENVs. Here we report the complete genome sequences of these three laboratory strains, dengue virus serotype-2 (DENV-2/KBPV-VR-29), -3 (DENV-3/KBPV-VR-30), and -4 (DENV-4/KBPV-VR-31) obtained from the Korea Bank for Pathogenic Viruses (http://kbpv.knrrc.or.kr) used for dengue virus research in South Korea.

Using a previously described protocol, each virus was reverse transcription (RT)-PCR amplified in five overlapping fragments that covered the entire polyprotein (4). Both sense and antisense strands of each fragment were sequenced at a commercial sequencing facility (Genotech, Daejeon, South Korea). The 5′ and 3′ terminal sequences of each viral genome were determined by rapid amplification of cDNA ends (RACE) using a FirstChoice RLM-RACE kit (Ambion, Austin, TX, USA) according to manufacturer’s instructions. The sequencing results were assembled using CodonCode Aligner v4.1.1 (CodonCode Inc., Centerville, MA, USA) and further analyzed using the Geneious 6.1.8 (Biomatters Ltd., Auckland, New Zealand) software.

The complete genome sequence of the DENV-2/KBPV-VR-29 was 10,712 nucleotides in length and encoded a 3,392-amino-acid polyprotein. Comparative genome analysis of DENV-2 showed nucleotide identities of 90.0% to 94.1% and amino acid identities of 96.0% to 97.8% compared to global DENV-2 strains available in GenBank. The complete genome sequence of DENV-3/KBPV-VR-30 was 10,696 nucleotides in length and encoded a 3,390-amino-acid polyprotein. Multiple sequence alignment of DENV-3/KBPV-VR-30 with complete genome sequences of global isolates of DENV showed 94.6% to 99.8% and 96.8% to 99.6% identity at the nucleotide and amino acid levels, respectively. Finally, we determined the complete nucleotide sequence of DENV-4/KBPV-VR-31. The genome was 10,664 nucleotides in length and encoded a 3,387-amino-acid polyprotein. Multiple sequence alignment of DENV-4/KBPV-VR-31 with full-length sequences of global isolates of DENV showed a range of 94.6% to 99.3% and 96.8% to 99.4% identities at the nucleotide and amino acid levels, respectively.

### Nucleotide sequence accession numbers.

The complete genome sequences of the DENV-2/KBPV-VR-29, DENV-3/KBPV-VR-30, and DENV-4/KBPV-VR-31 strains are deposited in GenBank under the accession numbers KP406804, KP406805, and KP406806, respectively.
